# Comparative Performance of Medical Students, ChatGPT-3.5 and ChatGPT-4.0 in Answering Questions From a Brazilian National Medical Exam: Cross-Sectional Questionnaire Study

**DOI:** 10.2196/66552

**Published:** 2025-05-08

**Authors:** Mateus Rodrigues Alessi, Heitor Augusto Gomes, Gabriel Oliveira, Matheus Lopes de Castro, Fabiano Grenteski, Leticia Miyashiro, Camila do Valle, Leticia Tozzini Tavares da Silva, Cristina Okamoto

**Affiliations:** 1 School of Medicine, Universidade Positivo, R. Prof. Pedro Viriato Parigot de Souza, 5300, Curitiba, 81280-330, Brazil, (41) 3317-3010

**Keywords:** artificial intelligence, intelligent systems, biomedical technology, medical ethics, exam questions, academic performance, AI, ethics, medical education, ChatGPT, medical exam, accuracy, medical student, observational study, medical data, medical school

## Abstract

**Background:**

Artificial intelligence has advanced significantly in various fields, including medicine, where tools like ChatGPT (GPT) have demonstrated remarkable capabilities in interpreting and synthesizing complex medical data. Since its launch in 2019, GPT has evolved, with version 4.0 offering enhanced processing power, image interpretation, and more accurate responses. In medicine, GPT has been used for diagnosis, research, and education, achieving significant milestones like passing the United States Medical Licensing Examination. Recent studies show that GPT 4.0 outperforms earlier versions and even medical students on medical exams.

**Objective:**

This study aimed to evaluate and compare the performance of GPT versions 3.5 and 4.0 on Brazilian Progress Tests (PT) from 2021 to 2023, analyzing their accuracy compared to medical students.

**Methods:**

A cross-sectional observational study was conducted using 333 multiple-choice questions from the PT, excluding questions with images and those nullified or repeated. All questions were presented sequentially without modification to their structure. The performance of GPT versions was compared using statistical methods and medical students’ scores were included for context.

**Results:**

There was a statistically significant difference in total performance scores across the 2021, 2022, and 2023 exams between GPT-3.5 and GPT-4.0 (*P*=.03). However, this significance did not remain after Bonferroni correction. On average, GPT v3.5 scored 68.4%, whereas v4.0 achieved 87.2%, reflecting an absolute improvement of 18.8% and a relative increase of 27.4% in accuracy. When broken down by subject, the average scores for GPT-3.5 and GPT-4.0, respectively, were as follows: surgery (73.5% vs 88.0%, *P*=.03), basic sciences (77.5% vs 96.2%, *P*=.004), internal medicine (61.5% vs 75.1%, *P*=.14), gynecology and obstetrics (64.5% vs 94.8%, *P*=.002), pediatrics (58.5% vs 80.0%, *P*=.02), and public health (77.8% vs 89.6%, *P*=.02). After Bonferroni correction, only basic sciences and gynecology and obstetrics retained statistically significant differences.

**Conclusions:**

GPT-4.0 demonstrates superior accuracy compared to its predecessor in answering medical questions on the PT. These results are similar to other studies, indicating that we are approaching a new revolution in medicine.

## Introduction

Artificial intelligence (AI) is a branch of science centered on the development of systems and algorithms capable of performing complex tasks that typically require human cognition. One well-known example is ChatGPT (GPT), introduced by OpenAI in 2019 [1]. Unlike other AIs, it relies on large language models and deep learning, which means that the tool uses vast amounts of text processed through deep neural networks to analyze and generate natural language with a high degree of complexity and precision, learning and evolving from its own mistakes. Its popularity stems from its ability to synthesize and interpret complex texts and respond to users within seconds. Instead of retrieving information directly from internet data sources, its responses are generated based on the probabilistic patterns of two or more words appearing together in a sentence. Since its launch, OpenAI has been making improvements and updating the tool, releasing GPT-v4.0 in March 2023, surpassing its predecessor, v3.5. The improvements include image interpretation, greater processing power, the ability to solve complex problems, interpretation of more words in a single query, and more accurate responses with improved understanding of the nuances of human language.

Since its development, AI has been used in various fields of medicine, with new studies focusing on medical education [2-5]. When GPT-3.0 was challenged with the United States Medical Licensing Examination (USMLE), it scored approximately 60%, enough to pass the exam, a milestone in the academic field, as the tool passed all three steps of an exam, which together contain more than 1,000 questions and are recognized worldwide for their difficulty. This performance suggested that GPT-3.0 had intelligence similar to that of a third-year medical student [6]. In addition, Liu et al [7] in 2024, conducted a meta-analysis evaluating the performance of GPT on 45 medical examinations worldwide. The study found that v4.0 achieved an overall accuracy rate of 81%, outperforming GPT-3.5, and in most cases, surpassing the average scores of medical students. In this study, 29 of the articles used v4.0, 26 tested the performance of v3.5, and 14 studies tested both GPT-4.0 and GPT-3.5. This study also found that translating the test into English significantly improved the accuracy of GPT-3.5, but not GPT-4.0. Only one Brazilian study was included in the meta-analysis, which reported 87.7% of correct answers for GPT-4.0 when exposed to the Brazilian National Examination for Medical Degree Revalidation (Revalida); however, only GPT-4.0 was evaluated in this study [8].

The present article builds upon the work of Rodrigues Alessi et al [9] in 2024, who applied GPT 3.5 to the Progress Tests (PT) of 2021, 2022, and 2023, finding an average accuracy of 68.4%, surpassing that of medical students from all years [9]. Although Brazil lacks a national medical exam for resident selection or for newly graduated doctors, the PT is a national exam in which over 50,000 medical students participated in recent editions. It consists of 120 multiple-choice questions, each with five alternatives and only a single correct answer, equally distributed across the areas of surgery, pediatrics, social sciences, internal medicine, basic sciences, and gynecology and obstetrics. In 2021, the Brazilian Association of Medical Education (ABEM) administered a single national exam, whereas in 2022 and 2023, the exams were conducted across different regions of Brazil. These last two evaluations have a regional scope, encompassing certain conglomerates of states in the country. One of these nuclei is the South II Institutional Pedagogical Support Center (NAPISUL II), which includes a total of 13 universities from Paraná and Santa Catarina. It is worth noting that this exam is administered by the same group, with the same number of questions and covering the same six topics. The difficulty and the manner in which the questions are evaluated are similar.

The exact evolution between GPT 3.5 and GPT-4.0 in answering Brazilian medical questions remains uncertain. Therefore, this study aimed to assess and compare the performance of medical students and the two GPT versions in answering questions from a Brazilian national medical exam in its native language.

## Methods

### Overview

The study had an observational, cross-sectional design to evaluate the performance of GPT 3.5 and GPT-4.0 on 333 questions from the 2021, 2022, and 2023 Progress Tests (PT) in its original language, excluding questions with images, nullified questions, and repeated questions. Each question was manually inputted into the AI platform, with the tool’s history cleared and session restarted after each question to avoid memory bias. Responses were categorized as correct or incorrect. In addition, the mean scores of first- through sixth-year medical students were compared using appropriate statistical methods to assess accuracy and effectiveness. Although ABEM did not release the number of students per year of medical school who participated in these tests, the approximate number of all tests together surpassed 50,000 medical students. All questions and the corresponding answer keys used in this study are publicly available.

### Inclusion and Exclusion Criteria

A total of 360 questions (120 from each test) from the 2021 National PT and the 2022 and 2023 Regional Tests (NAPISUL II) were included. We excluded questions that included images or figures containing graphs, questions that were repeated across the three tests evaluated, and questions that were invalidated by the test organizers (those found after the exam was administered to contain errors or issues such as ambiguous wording, incorrect answers, or flawed reasoning).

As a result, 333 multiple-choice questions were included: 109 from the 2021 PT, 117 from the 2022 NAPISUL II Test, and 107 from the 2023 NAPISUL II Test, each containing only one correct answer among five possible alternatives (A, B, C, D, or E).

### Procedures

First, each question included in the study criteria was submitted to the virtual platform GPT 3.5 and GPT-4.0 separately, and the responses were categorized into two possible outcomes, correct answer or incorrect answer, based on the official answer key for each test. To avoid memory bias, the platform’s history was deleted and the site was restarted after each question was presented.

In instances where the platform selected more than one answer to be correct, a follow-up question—“Which is the most correct alternative?”—was asked to obtain a single answer and improve statistical interpretation. If the new AI response matched the official answer key, it was considered correct; otherwise , it was considered incorrect.

Thus, three possible outcomes were obtained for each question for GPT: (1) correct; (2) initially incorrect but aligned with the answer key after it was presented; (3) incorrect and did not align with the answer key after it was presented.

The results were compared between GPT v3.5, v4.0, and the students, divided into overall average scores (ie, from students from the first to sixth year) and scores for only sixth-year students. The results were also analyzed into the six main subject areas of the test. For the 2021 test, the average percentage of correct answers per subject for students from the first to sixth year was not provided by ABEM, only the percentage per subject for sixth-year students was made public. Since the data were only available in averages, a statistical significance test could not be conducted.

### Data Evaluation

The data were organized in an Excel spreadsheet and analyzed using SPSS (version 29.0.0; IBM Corp) software. For descriptive analysis of quantitative variables, the mean, standard deviation, median, minimum, and maximum were presented. The Wilcoxon nonparametric test was used to compare the accuracy rates between GPT versions-3.5 and GPT-4.0, and Bonferroni corrections was performed for the comparisons. A *P* value <.05 was considered statistically significant.

### Ethical Considerations

This study only used information that was already publicly available on the internet and did not involve human subjects; rather, it was limited to an analysis of the PT performance. Therefore, approval by the institutional review board of Shimane University was not required.

## Results

### Main Findings

Based on the questions that met the inclusion criteria, our results showed that there is a statistically significant difference between the total performance scores of the three tests (2021, 2022, and 2023) between the GPT-3.5 and GPT-4.0 (*P* value .03). However, this difference lost significance after Bonferroni correction. The average accuracy for v3.5 was 68.4%, while v4.0 achieved an outstanding 87.2% accuracy rate. This indicates an absolute difference of 18.8% between the two versions and a relative improvement of 27.4% in accuracy in the latest platform version.

The mean scores by subject areas across the three tests were as follows for v3.5 and v4.0, respectively: 73.5% versus 88% for surgery (*P* value=.03), 77.5% versus 96.2% for basic sciences (*P* value=.004), 61.5% versus 75.1% for internal medicine (*P* value=.14), 64.5% versus 94.8% for gynecology and obstetrics (*P* value=.002), 58.5% versus 80% for pediatrics (*P* value=.02), and 77.8% versus 89.6% for public health (*P* value=.02) as shown in (Table 1). After Bonferroni corrections, only basic sciences and gynecology and obstetrics retained statistical significance.

The largest absolute difference in accuracy was observed in Gynecology and obstetrics (30.3%), corresponding to a relative improvement of 46.9%. Additionally, the smallest difference was in public health, with an absolute difference of 11.8% and a relative difference of 15.1%.

**Figure 1.**
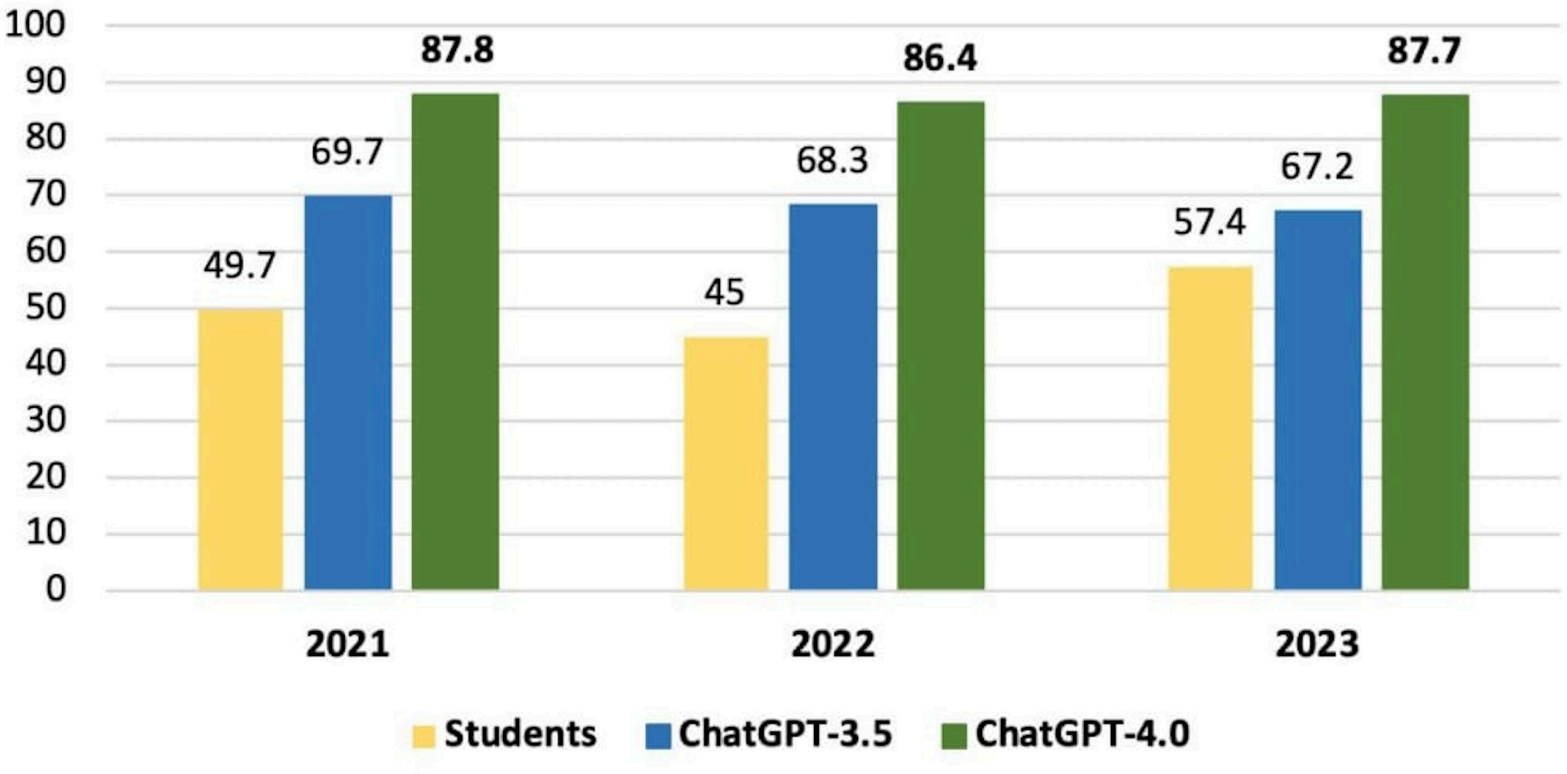
Comparison of performance accuracy of GPT-3.5, GPT-4.0, and medical students’ score by year.

Among the 333 questions selected for the study, the overall average accuracy of GPT-3.5 for the 2021, 2022, and 2023 tests was 69.7%, 68.3%, and 67.2%, respectively. On the other hand, GPT-4.0 scored 87.8%, 86.4%, and 87.7% for the same tests during that period. Conversely, medical students scored 49.7%, 45%, and 57.4%, respectively. Among the sixth-year medical students alone, the average accuracy was 66.3%, 56.5%, and 60% for the respective tests. These results are illustrated in Figures1  2.

In the 2021 test, due to the unavailability of students’ subject-specific accuracy data, it was not possible to compare students’ accuracy by subject against GPT. However, when comparing the overall accuracy scores, the scores were 49.7% for the students, 69.7% for GPT-3.5, and 87.8% for GPT-4.0. The values for GPT-3.5 and GPT-4.0, respectively, were as follows: 94.1% versus 94.1% (basic sciences), 68.7% versus 81.2% (surgery), 66.6% versus 66.6% (internal medicine), 50% versus 100% (gynecology and obstetrics), 59.0% vs 85.0% (Pediatrics), and 90.0% vs 100% (public health), as presented in Figure 3.

In the 2022 test, the overall scores were 45.2% for the students, 68.3% for GPT-3.5, and 86.4% for GPT-4.0. When evaluating the average accuracy per subject for students and GPT-3.5 and GPT-4.0, the results were as follows: 46.1% versus 73.6% versus 94.7% (basic sciences); 44% versus 68.4% versus 94.7% (surgery); 43.3% versus 65% versus 70% (internal medicine); 50.2% versus 68.4% versus 89.4% (gynecology and obstetrics); 42.3% versus 60% versus 80% (pediatrics); and 45.1% versus 75% versus 90% (public health), summarized graphically in Figure 4.

In 2023, the overall student score was 57.4%, compared to 67.2% for GPT-3.5 and 87.7% for GPT-4.0. The scores by subject for medical students, GPT-3.5 and GPT-4.0, respectively: 56.8% versus 64.7% versus 100% (basic sciences); 57.9% versus 83.3% versus 88.8% (surgery); 56% versus 52.9% versus 88.8% (internal medicine); 62.3% versus 75% versus 95% (gynecology and obstetrics); 56.9% versus 56.2% versus 75% (pediatrics); and 54.7% versus 68.4% versus 78.9% (public health), graphically illustrated in Figure 5.

The combined score analysis for the 2022 and 2023 tests, comparing medical students versus GPT-3.5 versus GPT-4.0, respectively was: 51.3% versus 67.8% versus 87% (overall); 51.5% versus 69.2% versus 97.3% (basic sciences); 51% versus 75.9% versus 91.7% (surgery); 49.7% versus 59% versus 79.4% (internal medicine); 56.3% versus 71.7% versus 92.2% (gynecology and obstetrics); 49.6% versus 58.1% versus 77.5% (pediatrics); and 49.9% versus 71.7% versus 84.4% (public health), as presented in Figure 6.

Data on average subject accuracy for sixth-year students in all three tests were made available by ABEM, which allowed to compare the accuracy of sixth-year students with GPT-3.5 and GPT-4.0. The results were as follows: 60.9% versus 68.4% versus 87.2% (overall); 54.8% versus 77.5% versus 96.2% (basic sciences); 51.2% versus 73.5% versus 88% (surgery); 53.8% versus 61.5% versus 75.1% (internal medicine); 56.7% versus 64.5% versus 94.6% (gynecology and obstetrics); 55.1% versus 58.5% versus 80% (pediatrics); and 55% versus 77.8% versus 89.3% (public health), as shown in Figures7  8.

The latest AI version (ie, GPT-4.0) answered two new incorrect questions and 12 new correct ones for basic sciences; four new incorrect questions and 11 new correct ones for surgery; four new incorrect questions and 11 new correct ones for internal medicine; two new incorrect questions and 18 new correct ones for gynecology and obstetrics; five new incorrect questions and 19 new correct ones for pediatrics; and two new incorrect questions and 10 new correct ones for public Health.

**Table 1.** Comparison of performance of GPT-3.5 versus GPT-4.0 based on the 6 test subjects and the overall score.

Subject	Version	Mean	*P* value^a^	*P* value^b^	Number of questions^c^
Surgery	v3.5	73.5	.03	.22	53
	v4.0	88.0		
Basic sciences	v3.5	77.5	.004	.03	53
	v4.0	96.2		
Internal medicine	v3.5	61.5	.14	.96	55
	v4.0	75.1		
Gynecology and obstetrics	v3.5	64.5	.002	.01	57
	v4.0	94.8		
Pediatrics	v3.5	58.5	.02	.15	56
	v4.0	80.0		
Public health	v3.5	77.8	.02	.15	59
	v4.0	89.6		
Overall	v3.5	68.4	.03	.20	333
	v4.0	87.2		

a Nonparametric Wilcoxon test was used and the statistical significance was defined as *P*<.05.

b Nonparametric Wilcoxon test with Bonferroni corrections was used and the statistical significance value was defined as *P*<.05.

c The number of questions from all three tests together is also present in the right column.

**Figure 2.**
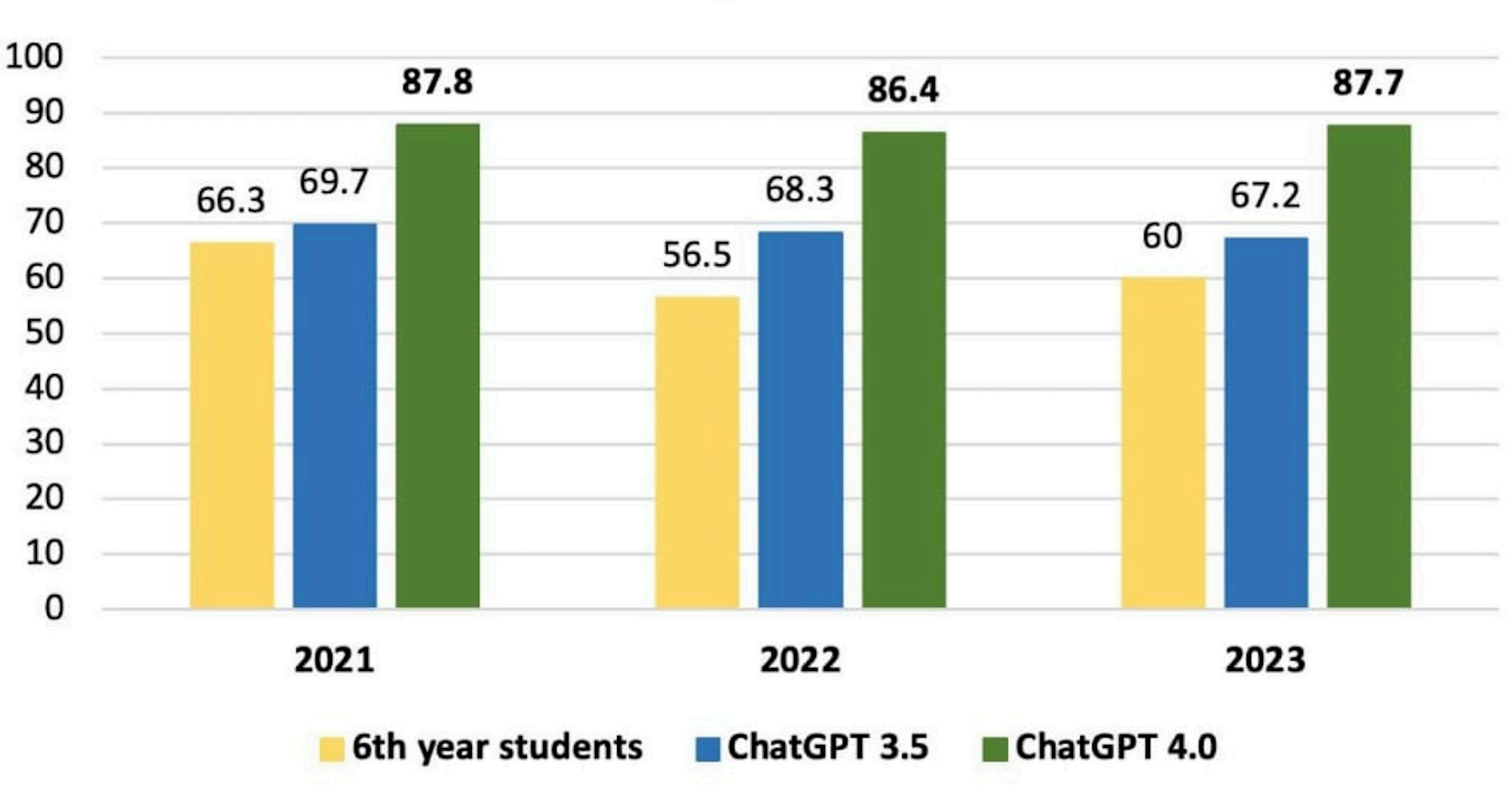
Comparison of performance accuracy of GPT-3.5, GPT-4.0, and sixth year medical students’ score by year.

**Figure 3.**
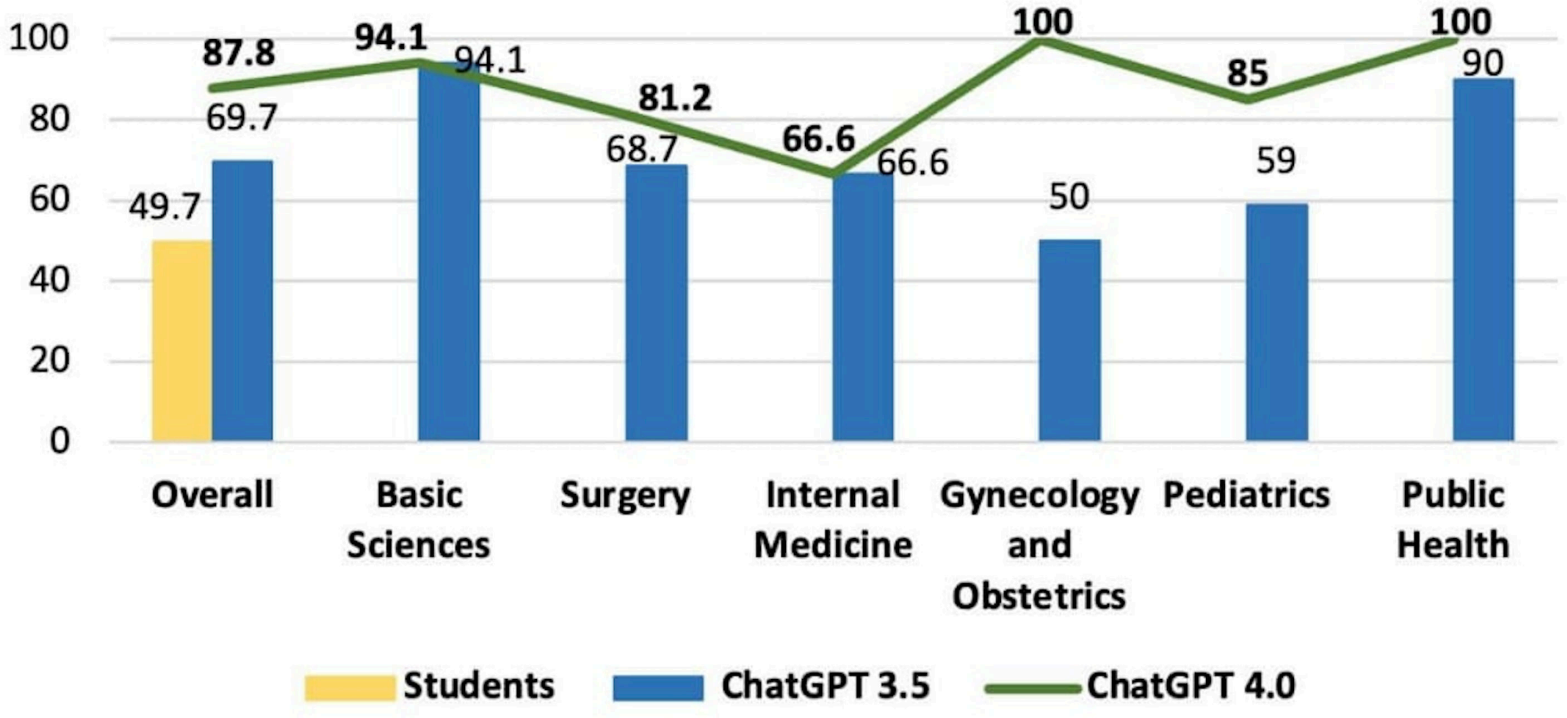
Comparison of overall accuracy scores between GPT-3.5, GPT 4.0, and medical students’ score in the 2021 progress test.

**Figure 4.**
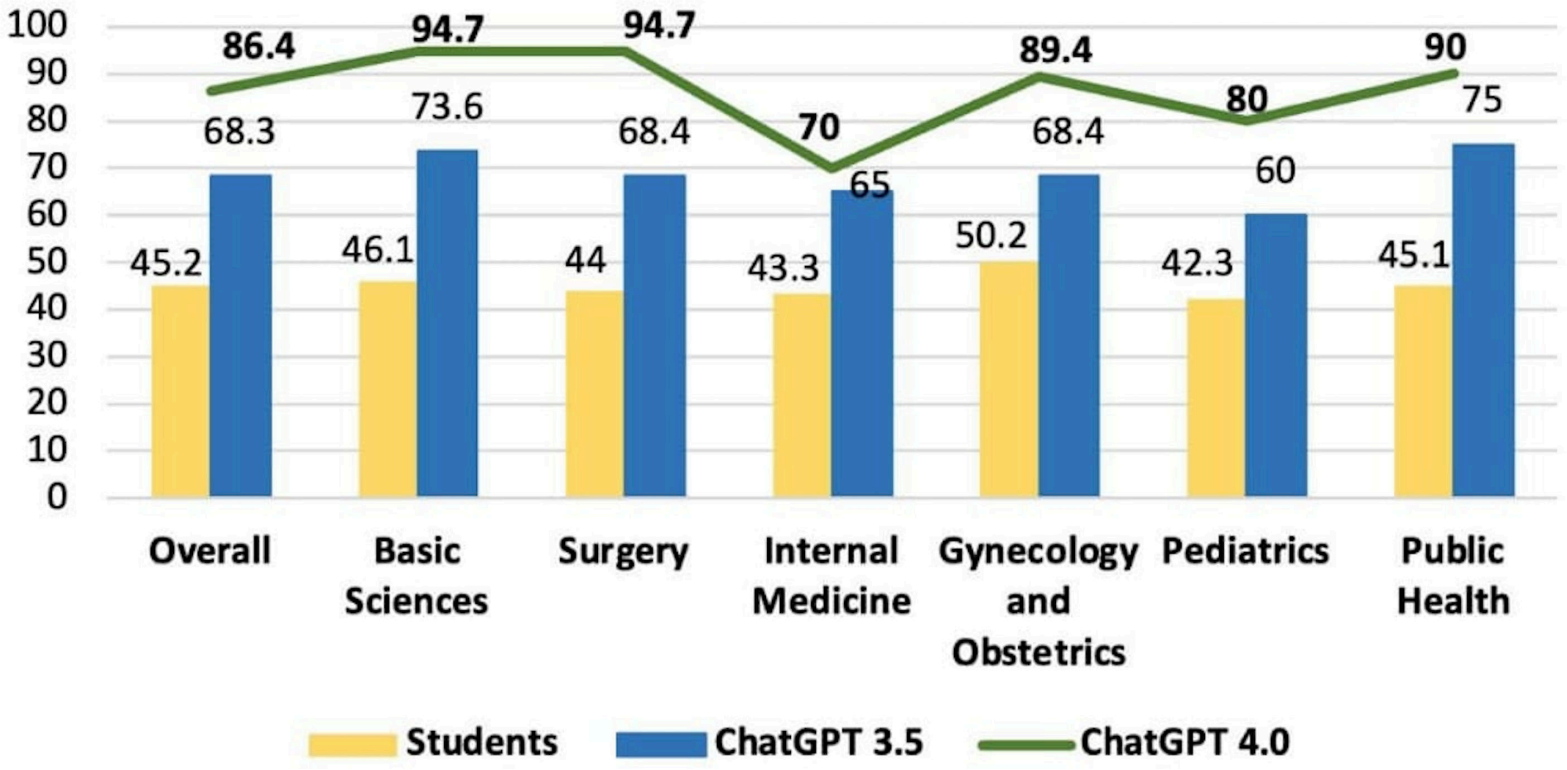
Comparison of overall accuracy scores between GPT-3.5, GPT-4.0, and medical students’ score in the 2022 progress test.

**Figure 5.**
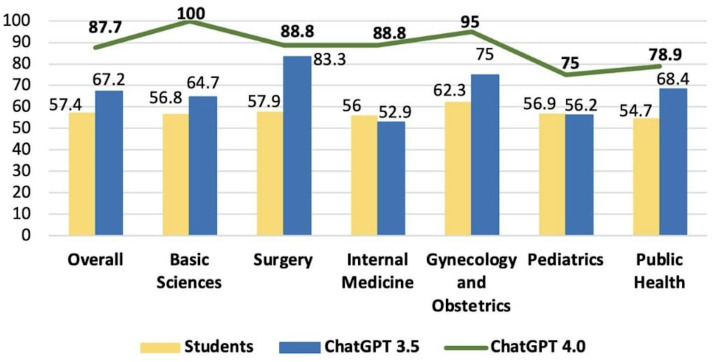
Comparison of overall accuracy scores between GPT-3.5, GPT-4.0, and medical student’s score in the 2023 progress test.

**Figure 6.**
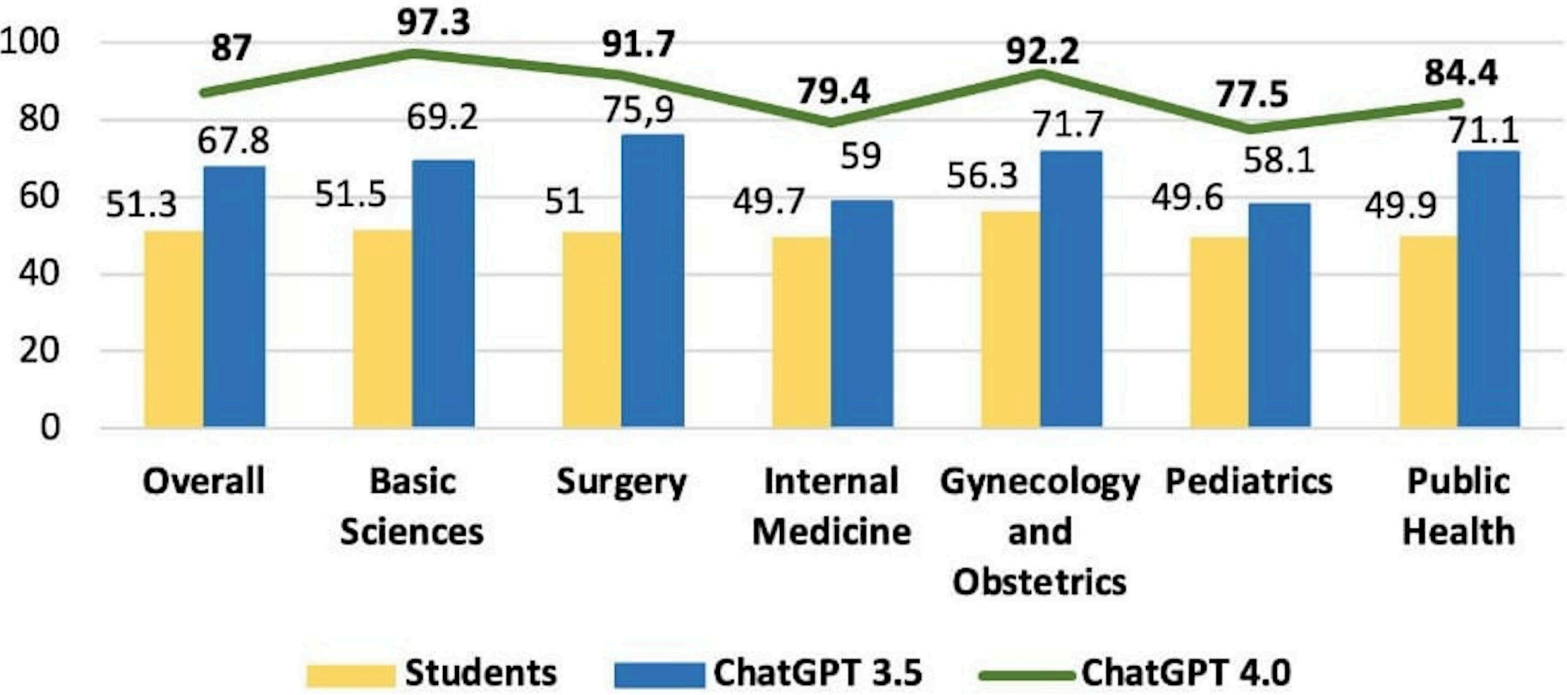
Comparison of GPT-3.5, GPT-4.0, and medical students’ combined scores in the 2022-2023 progress test.

**Figure 7.**
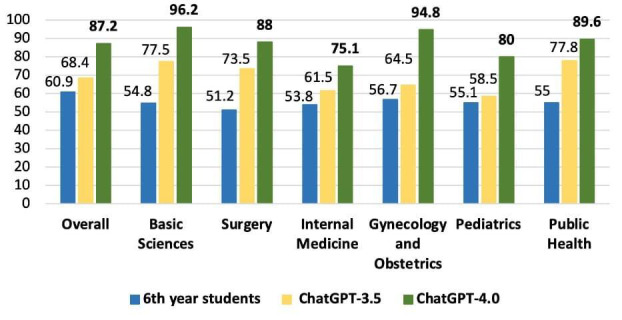
Comparison of GPT-3.5, GPT-4.0, and sixth-year medical students’ average subject accuracy score of the 2021-2023 progress test.

**Figure 8.**
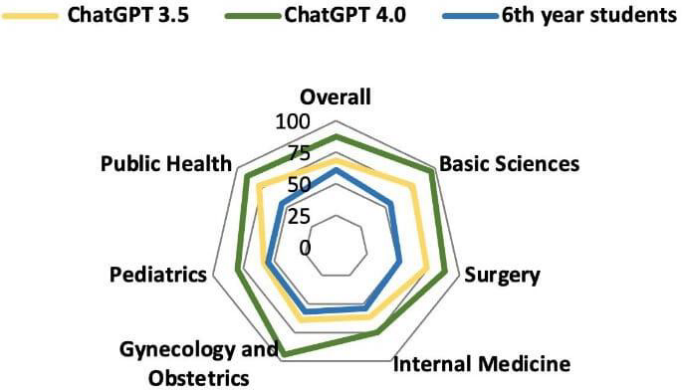
Comparison of GPT-3.5, GPT-4.0, and sixth-year medical student’s radar score in the 2022-2023 progress test.

### Questions Example and Responses

The answer key indicated that option C was the correct one, meaning GPT-4.0 selected the correct answer with a meaningful explanation, whereas GPT 3.5 made a mistake, but still tried to explain why it choose that alternative (Textbox 1).

Textbox 1. **Questions Example and Responses**

**Questions Example**

For demonstrative purposes, an example of a real question presented to GPT-3.5 and GPT-4.0 is presented below. All the text was inputted in Portuguese, and latter translated to English for better worldwide comprehension. However, it is important to highlight that all procedures in this research were conducted using the original language of the text.

Example questions: News reported that ovarian implantation technique can be successful in the field of fertility. A woman who suffered from leukemia had her ovaries removed and preserved in a laboratory; after undergoing treatment for the disease, she underwent reimplantation of the ovaries, successfully producing oocytes. After the medical-surgical procedure, it was possible to verify the growth and maturation of the oocytes. The hormones secreted by the pituitary gland and ovary, respectively, that induce ovulation are:

**Response Options**

- Follicle-stimulating and estrogen; luteotrophic hormone.
- Luteinizing and follicle-stimulating; progesterone.
- Stimulating and luteotrophic follicle; estrogens.
- Stimulating and luteotrophic follicle; progesterone.

GPT-3.5 answer: “The correct answer is option A: Follicle-stimulating and estrogens; luteotrophic hormone. Here is the explanation:

1. Follicle stimulating hormone (FSH): secreted by the pituitary gland, stimulates the growth and maturation of ovarian follicles, which contain oocytes. During this process, developing follicles secrete estrogens.
2. Estrogens: These are hormones produced by the ovaries (specifically by maturing follicles) and play a crucial role in regulating the menstrual cycle, promoting the growth of the endometrium, and preparing the body for a possible pregnancy.
3. Luteinizing hormone (LH) or luteotrophic hormone: It is also secreted by the pituitary gland and is responsible for triggering ovulation (the release of the mature oocyte from the follicle). After ovulation, LH promotes the formation of the corpus luteum, which secretes progesterone.

Therefore, ovulation is induced by pituitary hormones such as FSH, which stimulates the maturation of follicles and the production of estrogen and LH, which triggers ovulation.”

GPT-4.0 answer: “The correct answer is option C: Stimulating and luteotrophic follicle; estrogens. The explanation is given below:

- FSH: Hormone secreted by the pituitary gland, responsible for stimulating the growth and maturation of ovarian follicles, which contain oocytes.
- LH: It is also secreted by the pituitary gland and responsible for the ovulation process, where the mature follicle releases the oocytes.

- Estrogens: These are hormones produced by the ovary, important for the development and maintenance of the female reproductive system, and in regulating the menstrual cycle. They also play a crucial role in inducing the LH surge, which results in ovulation.

Therefore, the correct sequence is FSH and LH (luteotrophic), both from the pituitary, and estrogens from the ovary.”

## Discussion

### Principal Findings

GPT-4.0 displayed a high degree of superiority over its predecessor, improving its score by at least 10% across all subjects, with overall performance similar to those found in other studies. The average score of the three tests together was 87.2%, with a relative increase of 27.4% compared to the 3.5-version. Comparing the results obtained with those in the literature, which assessed the percentage of correct answers between the two GPT versions in various medical knowledge exams from different countries, it is noted that all studies found reported approximately 70% accuracy for GPT-4.0, with better performance by the new version and accuracy differences ranging from 5.34% to 32.1% [10-17]. A recent meta-analysis reviewing 45 studies that compared these two platforms in licensing examination tests across the globe found an accuracy rate above 80% for GPT-4.0, consistent with this study [7]. Like our results, Liu et al [7] in 2024 found many results surpassing human performance on the exams.

### Reflections

However, the question remains: is this improvement aligned with expectations? Analysts, including Bill Gates, have raised concerns that AI platforms such as GPT may be approaching a performance plateau, with each successive update yielding diminishing returns [18]. While there are no direct comparisons between GPT-2.5 or GPT-3.0 on PT questions, the leap from GPT-3.5 to GPT-4.0 was substantial, as evidenced by a 27.6% relative score increase observed in our study. Although this evolution does not necessarily mean that future updates will not encounter stagnation, it suggests that the plateau has not yet been reached. As further updates and studies emerge, further comparison across applications will be crucial.

While the results were impressive, the most important takeaway from this study is that GPT can be a reliable source for learning when used in conjunction with other tools. Its potential applications in medical education include communication, knowledge retention, writing and interpreting texts and images, individualized and personalized study, creation of clinical cases, and hypothesis generation for diseases, especially the rarer or overlooked conditions [19-23].

Despite all of these, some schools are working to restrict the use of AI in classrooms, fearing that students will misuse it to complete assignments faster without learning. This view is flawed since the focus should be on educating professors and institutions on how to integrate AI into curricula effectively. If a student can easily complete an assignment using GPT, then perhaps the task itself needs re-evaluation. Time and resources should not be spent on a task that AI can perform better This technology is too new, and professors are still learning how to use it; therefore, more time will be required to train teachers on balancing the use of AI in class. Ultimately, machine learning could prove as transformative as the internet revolution in academic settings.

In medicine, AI is already being used for the creation of medical forms, assistance in diagnoses and decision-making, interpretation and reporting of exams, patient monitoring, cost reduction, education, probabilistic prognostic elaboration, identification of treatment responses, medical record creation, among other applications [2-5]. However, we cannot overlook the potential concerns involving its use. The main barriers are validation, usability, utility, and ethics [24]. More global research is still needed to generate more data on the use of AI in medicine. Only after extensive proof of superiority will its use be legalized [25]. Utility refers to the functionality of a tool being studied and improved through research and its use in various functions, while usability refers to the ability of health care professionals to use the tool to achieve satisfactory results [25]. Finally, ethical considerations are a cornerstone in integrating AI in medicine [26]. AI systems can pose risks to privacy and confidentiality, as sensitive patient data could be vulnerable to misuse or unauthorized access. Additionally, biases inherent in the algorithms, such as those resulting from biased training data or model development, can perpetuate disparities in health care, potentially leading to unfair outcomes for certain groups. Furthermore, deploying AI in areas without prior validation can raise concerns about its reliability and safety in clinical decision-making, as well as its impact on patient care. These challenges underscore the importance of addressing ethical principles such as transparency, liability, and fairness in the development and application of AI in health care, ensuring it serves all patients equitably and responsibly.

Another key issue with GPT is the phenomenon by which the tool creates fictitious and incorrect information, referred to as “hallucinations” [27]. This action is not fully understood, but it results in a plausible-sounding, yet incorrect answer for the user, without informing the user of its inaccuracy [25]. The most concerning issue is that GPT can create detailed and local explanations that are completely wrong, yet may look very convenient for the reader. This phenomenon may explain why the AI answered some questions incorrectly despite previously scoring correctly.

There remain many unsolved questions. How will AI affect the doctor-patient relationship? Will the new generation of doctors be dependent on technology or will it enhance their abilities? What will become of medical intuition? These questions will only be answered with time, but the impact of a good patient-medical relationship in treating diseases or improving medication adherence must not be overlooked [28]. Additionally, we must consider the increasing dependence on technology in current practice. A physician should be able to diagnose appendicitis through physical examination, without requiring a computer tomography. The future generations of doctors must understand how to use technology to their advantage, without forgetting the basics of medicine. Finally, medical intuition has been used for years to assist health care professionals when machines could not detect abnormalities [29]. The instinct, or “sixth sense” developed with years of medical practice cannot be taught or ignored at the expense of algorithms and probability formulas. The central challenge will be integrating AI into medicine while preserving the essential human touch in patient care.

Although we are still in a period of study and adaptation regarding the implementation of these new technologies in medicine, it is evident that AI will replace humans in certain areas. Daily, new research is published daily presenting the use of machine learning in image interpretation, clinical reasoning, laboratory test analysis, drug development, and more [2-5,23,24]. Unfortunately, those who fail to stay updated with these advancements will be at greater risk of being left behind in the job market. Most likely, individual qualities will shift drastically toward more human characteristics such as empathy, creativity, abstraction, leadership, communication, and flexibility. Professionals who learn to work alongside new technologies, forming a human-machine symbiosis to take advantage of their functions while remaining empathetic and human, will likely be the most valued in the future.

### Limitations

Several limitations need consideration in this study such as the use of average student scores from the ABEM, which limits the ability to perform detailed statistical significance analyses. The AI’s last data update was in 2021, which may affect performance on more recent topics. Additionally, the optional nature of the PT introduces potential selection bias, as not all students or universities participate. Finally, LLMs can generate different responses each time due to inherent stochasticity, it is important to note that this variability was not systematically assessed in the present study and should be addressed in future research. Addressing these limitations in new studies may provide even more insightful results.

### Conclusions

GPT-4.0 demonstrates superior accuracy compared to its predecessor in answering medical questions on the PT. Its percentage of correct answers was higher across all subjects of the exam, even surpassing the scores of students from the first to sixth years of medical school. Although these findings are similar to previous studies, further research is required for the full implementation of AI in medicine. Nonetheless, it is evident that this technology is here to stay.

## Abbreviations

ABEM: Brazilian Association of Medical Education
AI: artificial intelligence
FSH: follicle-stimulating hormone
LH: luteinizing hormone
PT: progress test

## References

[1] ChatGPT (2024). https://chatgpt.com.

[2] Au K, Yang W (2023). Auxiliary use of ChatGPT in surgical diagnosis and treatment. Int J Surg.

[3] Hassan AM, Rajesh A, Asaad M (2023). Artificial intelligence and machine learning in prediction of surgical complications: current state, applications, and implications. Am Surg.

[4] Koohi-Moghadam M, Bae KT (2023). Generative AI in medical imaging: applications, challenges, and ethics. J Med Syst.

[5] Chenais G, Lagarde E, Gil-Jardiné C (2023). Artificial intelligence in emergency medicine: viewpoint of current applications and foreseeable opportunities and challenges. J Med Internet Res.

[6] Kung TH, Cheatham M, Medenilla A (2023). Performance of ChatGPT on USMLE: potential for AI-assisted medical education using large language models. PLOS Digit Health.

[7] Liu M, Okuhara T, Chang X (2024). Performance of ChatGPT across different versions in medical licensing examinations worldwide: systematic review and meta-analysis. J Med Internet Res.

[8] Gobira M, Nakayama LF, Moreira R, Andrade E, Regatieri CVS, Belfort R (2023). Performance of ChatGPT-4 in answering questions from the Brazilian National Examination for Medical Degree Revalidation. Rev Assoc Med Bras (1992).

[9] Rodrigues Alessi M, Gomes HA, Lopes de Castro M, Terumy Okamoto C (2024). Performance of ChatGPT in solving questions from the progress test (Brazilian National Medical Exam): a potential artificial intelligence tool in medical practice. Cureus.

[10] Takagi S, Watari T, Erabi A, Sakaguchi K (2023). Performance of GPT-3.5 and GPT-4 on the Japanese Medical Licensing Examination: comparison study. JMIR Med Educ.

[11] Rojas M, Rojas M, Burgess V, Toro-Pérez J, Salehi S (2024). Exploring the performance of ChatGPT Versions 3.5, 4, and 4 with vision in the Chilean Medical Licensing Examination: observational study. JMIR Med Educ.

[12] Lim ZW, Pushpanathan K, Yew SME (2023). Benchmarking large language models’ performances for myopia care: a comparative analysis of ChatGPT-3.5, ChatGPT-4.0, and Google Bard. EBioMedicine.

[13] Lee TJ, Rao AK, Campbell DJ, Radfar N, Dayal M, Khrais A (2024). Evaluating ChatGPT-3.5 and ChatGPT-4.0 responses on hyperlipidemia for patient education. Cureus.

[14] Choi J, Oh AR, Park J (2024). Evaluation of the quality and quantity of artificial intelligence-generated responses about anesthesia and surgery: using ChatGPT 3.5 and 4.0. Front Med (Lausanne).

[15] Taloni A, Borselli M, Scarsi V (2023). Comparative performance of humans versus GPT-4.0 and GPT-3.5 in the self-assessment program of American Academy of Ophthalmology. Sci Rep.

[16] Lechien JR, Briganti G, Vaira LA (2024). Accuracy of ChatGPT-3.5 and -4 in providing scientific references in otolaryngology–head and neck surgery. Eur Arch Otorhinolaryngol.

[17] Liang R, Zhao A, Peng L (2024). Enhanced artificial intelligence strategies in renal oncology: iterative optimization and comparative analysis of GPT 3.5 Versus 4.0. Ann Surg Oncol.

[18] Gates B Mit KI können medikamente viel schneller entwickelt werden [German]. Handelsblatt.

[19] Eysenbach G (2023). The role of ChatGPT, generative language models, and artificial intelligence in medical education: a conversation with ChatGPT and a call for papers. JMIR Med Educ.

[20] Sedaghat S (2023). Early applications of ChatGPT in medical practice, education and research. Clin Med (Lond).

[21] Lee H (2024). The rise of ChatGPT: exploring its potential in medical education. Anat Sci Educ.

[22] Mohammad B, Supti T, Alzubaidi M (2023). The pros and cons of using ChatGPT in medical education: a scoping review. Stud Health Technol Inform.

[23] Hunter DJ, Holmes C (2023). Where medical statistics meets artificial intelligence. N Engl J Med.

[24] Hamet P, Tremblay J (2017). Artificial intelligence in medicine. Metab Clin Exp.

[25] Sallam M (2023). ChatGPT utility in healthcare education, research, and practice: systematic review on the promising perspectives and valid concerns. Healthcare (Basel).

[26] Borenstein J, Howard A (2021). Emerging challenges in AI and the need for AI ethics education. AI Ethics.

[27] Dave T, Athaluri SA, Singh S (2023). ChatGPT in medicine: an overview of its applications, advantages, limitations, future prospects, and ethical considerations. Front Artif Intell.

[28] Diamond-Brown L (2016). The doctor-patient relationship as a toolkit for uncertain clinical decisions. Soc Sci Med.

[29] Duarte-Rojo A, Sejdic E (2022). Artificial intelligence and the risk for intuition decline in clinical medicine. Am J Gastroenterol.

